# Collective dynamics of *Escherichia coli* growth under near-lethal acid stress

**DOI:** 10.1128/mbio.01932-25

**Published:** 2025-09-22

**Authors:** Rafael R. Segura Munoz, Victor Sourjik

**Affiliations:** 1 Max Planck Institute for Terrestrial Microbiology 28310, Marburg, Germany; 2 Center for Synthetic Microbiology (SYNMIKRO), Marburg, Germany; Gulbenkian Institute for Molecular Medicine, Oeiras, Portugal

**Keywords:** *Escherichia coli*, Enterobacteriaceae, acid resistance, collective behavior

## Abstract

Many neutralophilic bacteria, including *Escherichia coli*, can withstand acidic conditions due to the action of several protective mechanisms. While the survival of *E. coli* under growth-inhibitory extreme acid stress is well understood, less is known about the physiology and ecology of *E. coli* under severe but growth-permissive acidity. Here, we observed that growth of *E. coli* MG1655 in a rich medium with a pH value of 4.1–4.4 exhibits a characteristic multiphasic growth pattern, which consists of an initial exponential elongation, up to 10 times the normal cell length, in the absence of cell division, followed by growth arrest and subsequent resumption of exponential growth. The duration of the growth arrest phase was strongly dependent on the pH of the medium, but also on the initial cell density of the culture, suggesting the collective nature of this phenomenon. Cell density-dependent multiphasic growth at the near-lethal pH, including the initial increase in cell volume associated with either elongation or widening of the cell body, was also observed for all tested natural *E. coli* isolates and for *Salmonella enterica* serovar Typhimurium. A transient increase in volume apparently enables bacteria to induce acid resistance systems, primarily the lysine-dependent Cad system, to subsequently modify the pH of the medium in the density-dependent manner. Consistent with the collective recovery, even a minor fraction of acid-tolerant cells could cross-protect acid-sensitive cells, enabling them to resume growth in coculture. Thus, collective dynamics plays an important role in bacterial growth under near-lethal acid stress and has potentially important ecological consequences.

**IMPORTANCE:**

Since many *E. coli* and other Enterobacteriaceae isolates are gastrointestinal pathogens for humans, it is important to understand their growth under acidic conditions imposed by the host physiology in the stomach, colon, and inside macrophages. Here we show that *E. coli* growth under near-lethal acidic conditions is a collective phenomenon that critically depends on cell density and on acid-resistance systems and is favored by changes in cell morphology as a response to high acidity. This collective behavior enables cross-protection between bacteria in microbial communities, and it might have implications for pathogen proliferation in the acidic environment of the human gastrointestinal tract and possibly also for their interactions with immune cells.

## INTRODUCTION

Neutralophilic bacteria grow optimally about two units below and above pH 7.0 (1). Some neutralophiles, such as *Escherichia coli*, have evolved multiple protective mechanisms that allow them to grow in acidic environments, such as the mammalian gastrointestinal tract and acidified water, soil, and food (2–8). The acid resistance (AR) of *E. coli* and other human pathogens has mostly been studied under growth-inhibiting, lethal acid stress (pH <3.0) that simulates an infection-related passage through the stomach (9, 10) and, to a much lesser extent, under growth-permitting, near-lethal acid stress (pH 4.0–5.0) that mimics other common acidic environments (9, 11), such as the human duodenum, duodeno-jejunal junction, and proximal colon (7, 8).

Among multiple protective mechanisms of *E. coli*, the most prominent are the amino acid-dependent AR systems, which consume H^+^ via amino acid decarboxylation to raise the cytoplasmic pH (2–5). These include the glutamate-dependent Gad system (AR2), the arginine-dependent Adi system (AR3), and the lysine-dependent Cad system (AR4) (2, 12). Each of these systems consists of a pH-dependent transcriptional regulator (GadE, AdiY, or CadC, respectively) and an amino acid decarboxylase (GadA/GadB, AdiA, or CadA) that sequesters intracellular H^+^ during the production of either γ-aminobutyric acid (GABA), agmatine, or cadaverine, respectively. Then, these biogenic amines are exported by an antiporter (GadC, AdiC, or CadB), which at the same time imports the corresponding amino acid for the AR system. These systems are known to be induced under different conditions: the Gad and Adi systems were reported to be particularly important under extreme acid stress, stationary phase, and/or anaerobic conditions, whereas the Cad system operates under mild (pH 5.0–5.8) and near-lethal acid stress (13). Moreover, a recent study demonstrated that operation of these systems may be partitioned within the same population of *E. coli* cells, with only fractions of cells expressing the Cad or Adi systems (13, 14). Such heterogeneity in the induction of stress response pathways indicates bet-hedging behavior, but possibly also division of labor and potential for collective behaviors in bacterial populations under acidic pH (13). Population density has indeed been shown to affect *E. coli* survival after exposure to lethal pH 2 (10, 15), but the nature of this effect remained unknown.

Here we show that, upon sudden exposure to near-lethal pH, strains of *E. coli* as well as *Salmonella enterica* serovar Typhimurium exhibit cell density-dependent multiphasic growth. In *E. coli* K-12, it consists of the initial phase of exponential elongation in the absence of cell division, followed by a transient cessation and a subsequent recovery of normal growth. The duration of this transient growth arrest was observed to be strongly dependent on cell density, apparently due to the collective deacidification of the medium during this phase that is largely dependent on the Cad system. The initial phase of the cell size increase was apparently important to enable the biosynthesis of the acid resistance systems, and it was also observed in other isolates of *E. coli* and *S*. Typhimurium. Consistent with the collective deacidification, we observed that even a minor proportion of acid-tolerant *E. coli* cells is sufficient to support growth of the acid-sensitive *E. coli* population at near-lethal pH.

## RESULTS

### *E. coli* exhibits density-dependent multiphasic growth under severe acid stress

**Fig 1:**
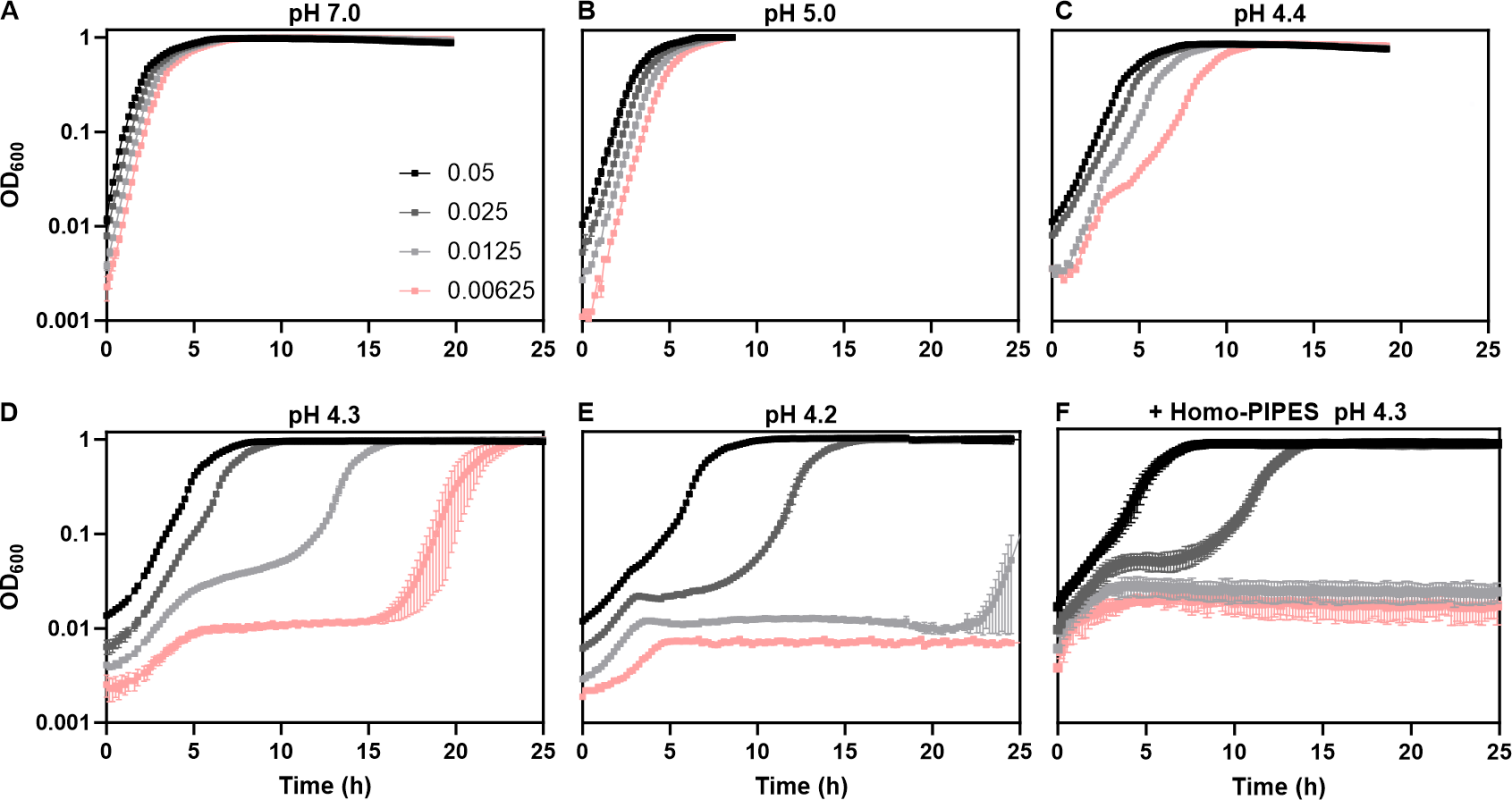
*E. coli* exhibits density-dependent growth at low pH. Growth of *E. coli* MG1655 measured as optical density at 600 nm (OD_600_) using plate reader at indicated inoculum sizes in unbuffered LB adjusted to an initial pH of (**A**) 7.0, (**B**) 5.0, (**C**) 4.4, (**D**) 4.3, (**E**) 4.2, or in (**F**) LB buffered with 33 mM homo-PIPES at pH 4.3. Inoculum size values indicated in panel **A** and throughout the paper correspond to OD_600_ measurements in a 10 mm cuvette using a spectrophotometer, thus deviating from OD_600_ values measured in a plate reader. Mean and SEM of three biological replicates are shown.

To investigate the growth dynamics of *E. coli* at different values of acidic pH, we inoculated cultures at different inoculum sizes in lysogeny broth (LB) medium with initial pH ranging from 7.0 to 4.0. Pre-culture of *E. coli* cells harvested at late exponential phase in LB at pH 7.0 was used for inoculation (see Materials and Methods) to ensure that cells can potentially resume growth directly after being transferred to the new medium. We observed that the growth rate of *E. coli* K-12 strain MG1655 decreased progressively at a lower pH (Fig. 1A through E; Fig. S1). Although the growth rate was apparently independent of the cell culture density at pH 7.0 or 5.0 (Fig. 1A and B), highly pronounced cell-density dependence of growth was observed starting at pH 4.4. Specifically, bacterial cultures exhibited multiphasic growth, including an initial, apparently exponential phase of increase in optical density at 600 nm (OD_600_), followed by an intermediate lag (mid-lag) phase and then a second exponential growth phase. At pH 4.4, such multiphasic growth was only observed for the lowest density culture (Fig. 1C), whereas high cell-density cultures showed continuous growth. At decreasing pH, the multiphasic growth became increasingly pronounced also for intermediate- and high-density cultures (Fig. 1D and E; Fig. S1A and B). No growth was observed at pH 4.0 (Fig. S1C). Importantly, the duration of the growth arrest phase showed strong dependence not only on pH but also on the cell density, with higher-density cultures exhibiting proportionally shorter or no lag phase.

We further investigated whether the density-dependent multiphasic growth is affected by the buffering capacity of the medium. We observed that the density dependence of growth shifted for *E. coli* cultures grown in LB with pH 4.3 but buffered with 33 mM homopiperazine-1,4-bis-2-ethanesulfonic acid (homo-PIPES), with higher-density cultures in buffered LB showing growth similar to lower-density cultures in non-buffered LB (Fig. 1F). Moreover, culture growth at pH 4.3 entirely ceased when LB was buffered with 66 mM homo-PIPES (Fig. S2).

### Density-dependent growth is characterized by cell elongation and medium deacidification

**Fig 2:**
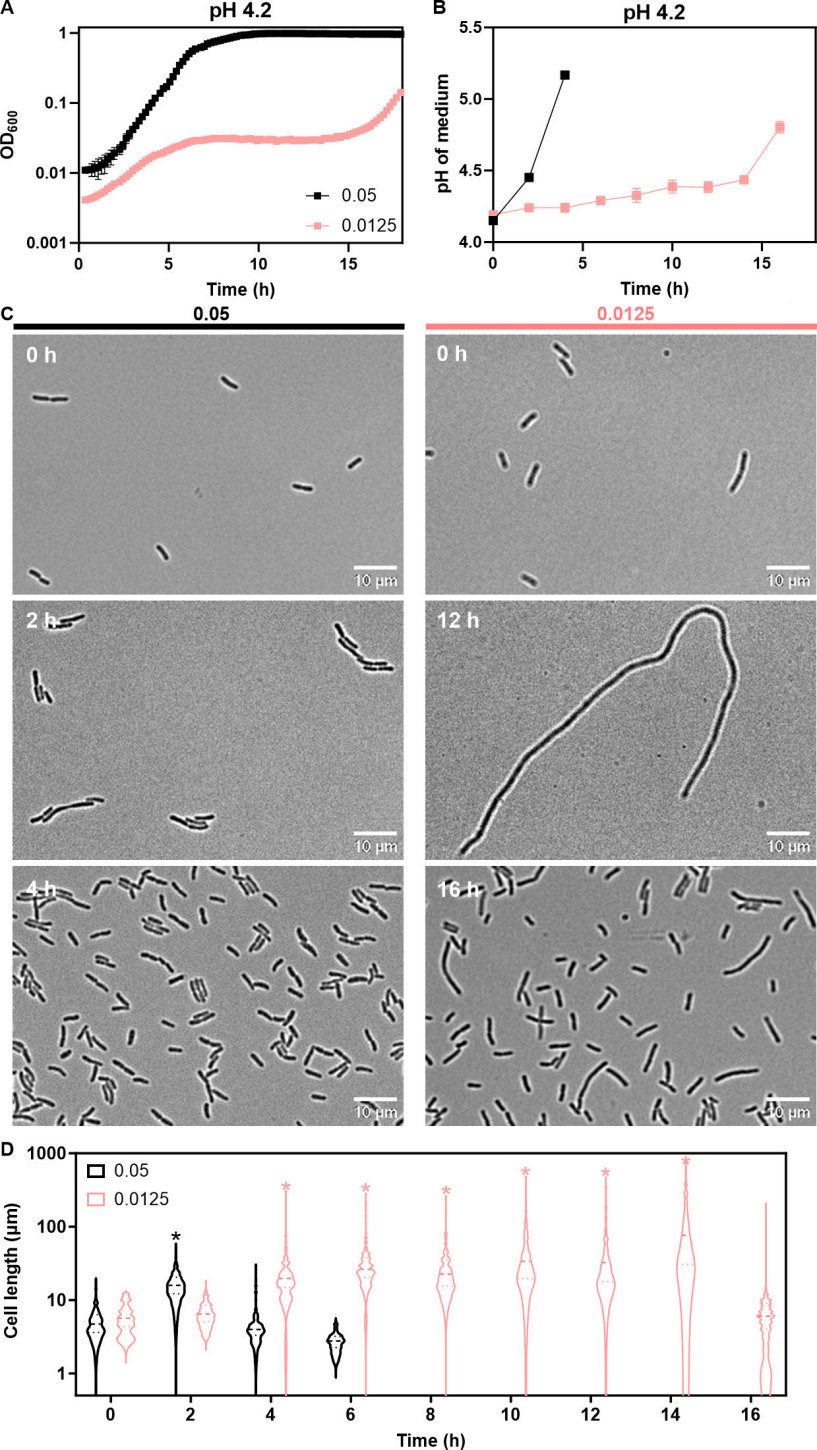
Multiphasic *E. coli* growth reflects changes in cell size and medium deacidification. *E. coli* MG1655 cultures were grown in LB at pH 4.2. (**A**) Growth of the cultures with indicated inoculum sizes, measured using a plate reader. (**B**) Medium pH for the same cultures, measured as described in Materials and Methods. Mean and SEM of three biological replicates are shown. pH values of cultures at the end of the experiment were 8.4 ± 0.1 (inoculum size 0.05) and 8.3 ± 0.1 (inoculum size 0.0125). (**C**) Morphology of *E. coli* cells in cultures with different inoculum sizes at indicated time points. Scale bar is 10 µm. (**D**) Quantification of cell length for 100–150 cells at indicated time points, indicating the distribution, the average (dashed line), and the upper and lower quartiles (dotted lines). Significance of difference compared to the time point zero, assessed using a paired *t*-test, is indicated by asterisks (*P ≤ 0.05).

Given the observed effects of buffering, we hypothesized that medium deacidification may dictate the observed multiphasic growth. Consistently, the final pH of all cultures that reached high optical density (OD) increased to >8.0, irrespective of their initial pH, while that of non-growing cultures remained low ( Table S1). To elucidate how deacidification of the medium correlates with the multiphasic growth, OD and medium pH were monitored simultaneously for *E. coli* cultures with different inoculum sizes growing at an initial pH of 4.2 (Fig. 2; Fig. S3). We observed that changes in pH were consistent with the cell growth (Fig. 2A and B; Fig. S3A and B). In particular, for low-density cultures where the multiphasic growth was pronounced, the exit from the intermediate lag phase coincided with the increase in pH. This resumption of cell growth occurred at pH ~4.4, and it was preceded by a very slow gradual elevation of pH over the entire duration of the initial phase of OD increase and the mid-lag phase. In contrast, the high-density culture crossed this apparently critical pH value already during the first phase and thus did not enter the intermediate phase.

To understand the nature of the initial phase of largely density-independent OD increase, we investigated changes in cell morphology during different growth phases using microscopy. In all cultures growing at pH 4.2, cells showed pronounced but transient elongation, with its extent and the duration of the elongation phase dependent on the inoculum density (Fig. 2C and D; Fig. S3C). We thus concluded that this up to ~10-fold increase in OD during the initial, apparently exponential growth phase reflects the elongation of cells from the initial average cell length of 4.4 to ~50.0 µm, in the absence of cell division. Consistently, there was no increase, and even a moderate decrease, in the number of colony-forming units (CFUs) in the culture during the initial growth phase (Fig. S4). This confirms that the observed increase in OD is indeed due to the cell elongation rather than the increase in the number of cells. Moreover, since the viability of these elongated cells is apparently partly compromised, there might be some release of their cytoplasmic content due to cell bursting. But although the release of the more neutral cytoplasmic content could contribute to deacidification of the medium, such contribution is likely to be minor, given low cell densities in our experiments. The reduction in the cell length subsequently occurred concurrently with the resumption of growth and increase in the CFU number after the mid-lag phase.

Since the increase in the cell length reproducibly saturated during the initial elongation phase, at the population average of ~50 µm (Fig. 2D; Fig. S3C), we hypothesized that this apparent limit to elongation may be due to a maximum of length that could be reached by *E. coli* cells. To test this, we incubated *E. coli* in LB at pH 7 with a known inhibitor of cell division, cephalexin, and monitored their elongation over time (as OD increase, Fig. S5A). The rate of cell elongation induced by cephalexin at this neutral pH was faster than that induced at near-lethal acidic pH, but the elongation saturated at nearly the same average cell length (Fig. S5B). This observation agrees with our hypothesis that the maximal increase of OD at near-lethal acidic pH is due to the limit on cell elongation.

Finally, we investigated whether the inhibited growth of *E. coli* culture during the mid-lag phase at near-lethal acidic pH might lead to emergence of acid-tolerant *E. coli* mutants. For that, *E. coli* cells were harvested after a passage at pH 4.2 upon inoculation at low culture density, and this culture was subsequently compared for its ability to grow at near-lethal acidic pH to the original culture (Fig. S6). This pre-exposed *E. coli* culture showed the multistage growth similar to that of the original culture, but it exited the mid-lag phase slightly faster. This suggests that selection for mutations that mildly increase acid tolerance might be indeed taking place in the culture during the prolonged mid-lag phase, but these mutations do not abolish the mid-lag phase.

### Pre-induced non-growing cells are able to deacidify the medium

**Fig 3:**
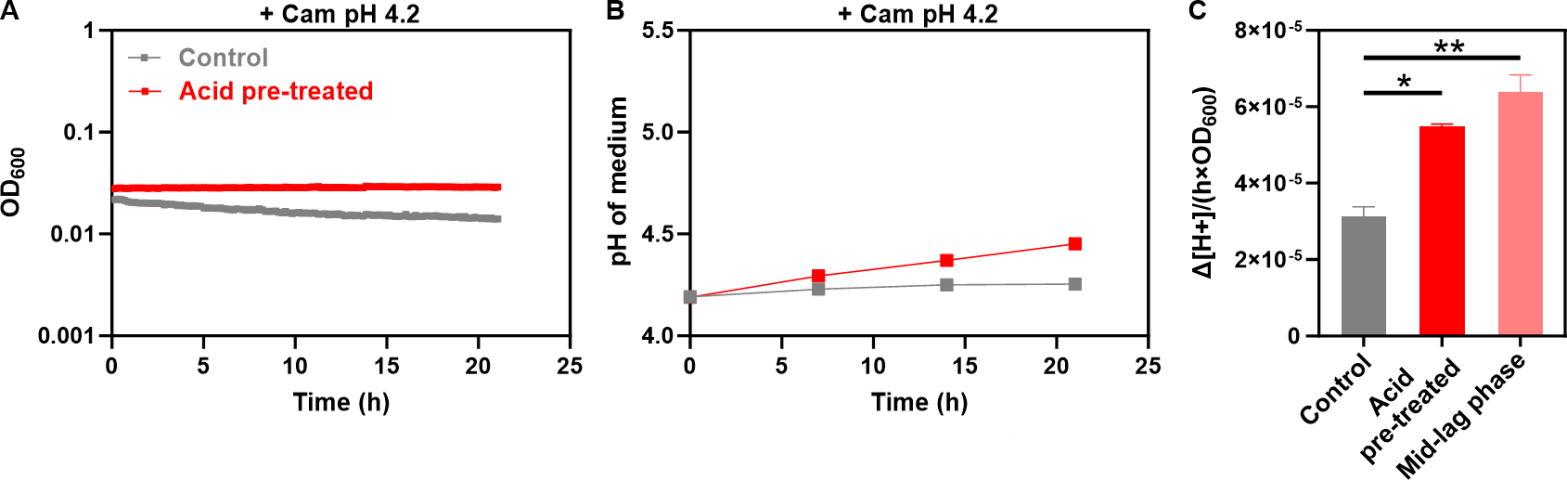
Pre-induced non-growing cells can efficiently deacidify the medium. *E. coli* MG1655 cells treated with chloramphenicol (Cam) to inhibit translation were incubated in LB at pH 4.2. Inoculum size was 0.1. (**A**) Density of the culture. (**B**) Medium pH. Prior to the Cam treatment, cells were exposed to either pH 4.2 (acid pre-treated) or pH 7.0 (control) for 1 h in LB. (**C**) Rate of deacidification, calculated as the molar amount of protons neutralized per OD_600_ per hour in translation-inhibited cells, or in the cell culture without translation inhibition during the mid-lag phase of growth at pH 4.2 (data from Fig. 2B). Mean and SEM of three biological replicates are shown. Significance of differences, assessed using an unpaired *t*-test, is indicated by asterisks (*P ≤ 0.05, **P ≤ 0.01).

Our results indicate that the density dependence of growth at pH below 4.4 could be explained by gradual deacidification of the medium over the net duration of the initial elongation and subsequent mid-lag phase, until the critical permissive pH above 4.4 is reached and the growth/division arrest is relieved. The ability of non-growing cells to deacidify the medium was further confirmed by monitoring pH in a culture of translation-inhibited cells (Fig. 3). Here we compared *E. coli* cultures that were pre-grown in LB at either neutral or acidic (4.2) pH for 1 h and subsequently transferred to LB containing chloramphenicol at pH 4.2 for 3 h to inhibit translation. The density of both cultures was adjusted to OD_600_ = 0.1 to reflect the cell density at the mid-lag phase of the 0.0125 inoculum culture (Fig. 2A), and OD_600_ and medium pH were monitored. We observed that translation-inhibited cells pre-incubated in acidic medium did not grow but increased medium pH to approximately 4.4 in 14 h (Fig. 3A and B). This rate of deacidification, calculated per hour and per unit of OD_600_, was similar to that observed during the mid-lag phase of growing cultures (Fig. 2B and 3C). The culture that was not pre-exposed to acidic pH prior to the inhibition of translation deacidified the medium less efficiently, suggesting that the growth-dependent pre-induction of the AR system(s) plays an important role in the subsequent deacidification of the medium by non-growing cells.

### Importance of individual AR systems in multiphasic growth under acid stress

**Fig 4:**
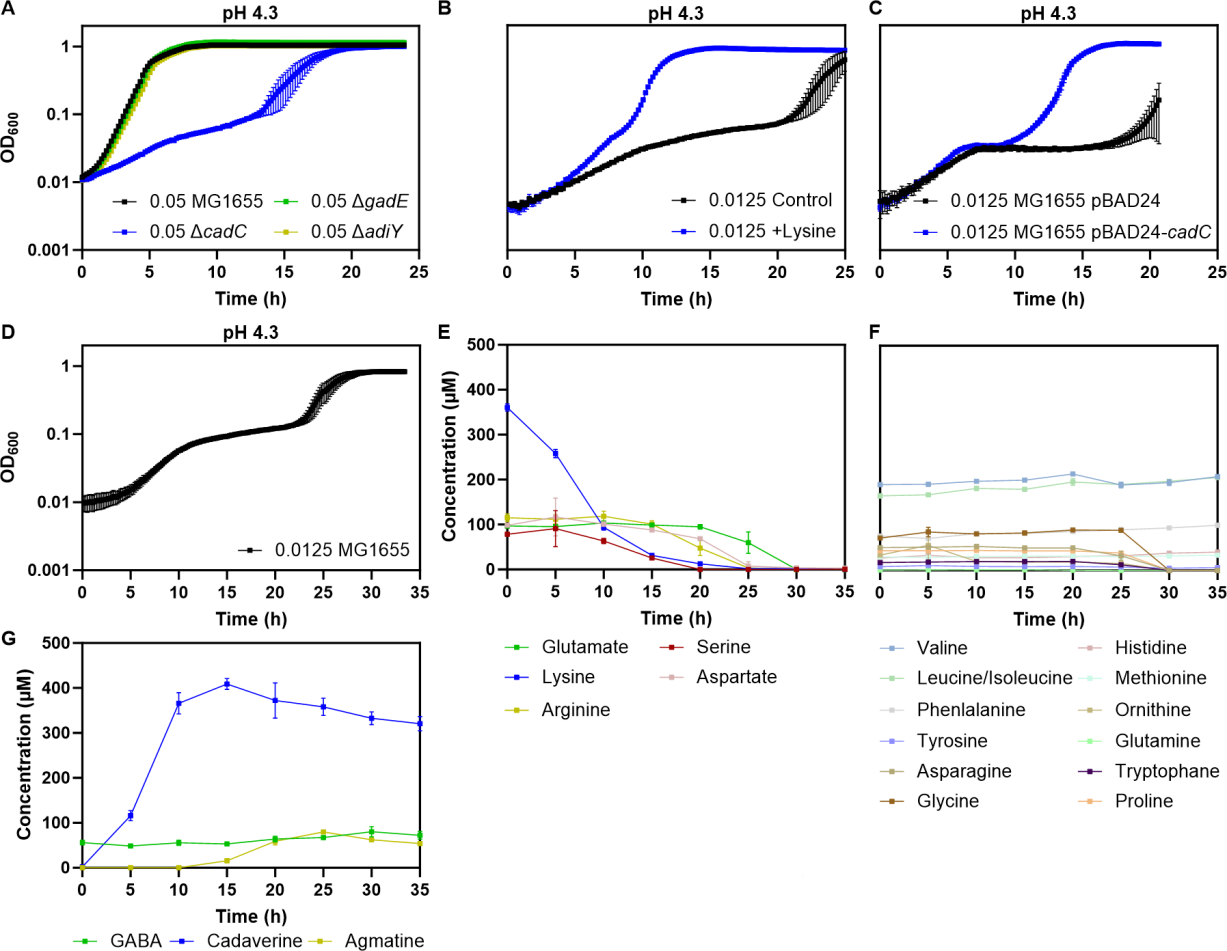
Cad system is primarily involved in cell density-dependent acid tolerance. (**A**) Growth of *E. coli* MG1655 wild type and of Δ*cadC*, Δ*gadE*, or Δ*adiY* mutants, as indicated, in LB at pH 4.3. Inoculum size was 0.05 for all strains. (**B**) Growth of *E. coli* MG1655 in LB with or without 20 mM lysine supplementation, at inoculum size of 0.0125. (**C**) Growth of *E. coli* MG1655 carrying either pBAD24 or pBAD24-*cadC*, at inoculum size of 0.0125. Although expression of the pBAD24-*cadC* construct is inducible by L-arabinose, no arabinose was added in this experiment as the uninduced expression was already sufficiently high to observe the effect. (**D–G**) Quantification of amino acids and biogenic amines during growth of wild-type *E. coli* MG1655 in LB, pH 4.3, at inoculum size of 0.0125. Shown are OD_600_ (**D**), levels of indicated amino acids that were either at least partly consumed (**E**) or remained unaltered (**F**) during the mid-lag phase, and levels of indicated biogenic amines (**G**) in the medium. Mean and SEM of three biological replicates are shown.

To assess which AR system(s) contributes to the acid tolerance of *E. coli* during multiphasic growth under near-lethal acid stress, we first tested *E. coli* strains deficient in the expression of individual AR systems. Strain lacking *cadC* showed much reduced growth in LB pH 4.3, with lower initial rate of OD increase and a pronounced mid-lag phase even at high inoculum size (Fig. 4A). Consistent with this, both supplementation of LB pH 4.3 with lysine (Fig. 4B; Fig. S7) and the overexpression of CadC (Fig. 4C) resulted in a strong improvement in growth at low initial cell density, with a faster initial increase in OD and a reduced duration of the mid-lag phase. No growth defects comparable to that of Δ*cadC* were observed for strains deleted for genes encoding the transcriptional activators of AR2 (Δ*gadE*) or AR3 (Δ*adiY*) systems at pH 4.3 (Fig. 4A), suggesting that these systems play at most auxiliary roles. Nevertheless, a moderate reduction of the initial growth was observed in the Δ*gadE* strain at the even lower pH 4.2 (Fig. S8A), and the overexpression of GadE had a positive effect on growth and shortened the mid-lag phase (Fig. S8B).

We further investigated changes in the levels of amino acids and biogenic amines in the medium during *E. coli* MG1655 growth in LB at pH 4.3 (Fig. 4D). During the pre- and mid-lag phases, lysine was the most prominently consumed amino acid (Fig. 4E). This is in stark contrast to *E. coli* growth at neutral pH, where no consumption of lysine by MG1655 was observed (16), which supports the key function of the AR4 (Cad) system in the deacidification of the medium. Arginine was consumed during the second half of the mid-lag phase, demonstrating that the AR3 system is also active. Consistent with such consumption of AR substrates, cadaverine was the primary biogenic amine produced already during the initial growth and even more prominently during the mid-lag phase, while the production of agmatine was only observed later in the mid-lag phase (Fig. 4G). Glutamate was only consumed after the restoration of growth (Fig. 4E), and GABA levels remained unchanged during the pre- and mid-lag phases (Fig. 4G). Besides these amino acid substrates of the acid resistance systems, we also observed consumption of serine and, to a lesser extent, aspartate during the mid-lag phase. These amino acids are those most efficiently metabolized during *E. coli* growth at the neutral pH (16), indicating that their consumption during the mid-lag phase may not be related to the acid stress protection. Nevertheless, it might also be related to a recently described mechanism of acid tolerance that depends on serine deamination (17). All other detected amino acids were consumed exclusively during the restoration of growth or remained unaltered throughout the multiphasic growth (Fig. 4F). These data further confirm that the AR4 (Cad) system is the main contributor to both the initial growth resulting in cell elongation and the exit from the mid-lag phase, while the other AR systems make lesser contributions under these conditions.

### Other *E. coli* and *Salmonella* strains exhibit cell density-dependent growth and morphological changes at near-lethal pH

**Fig 5:**
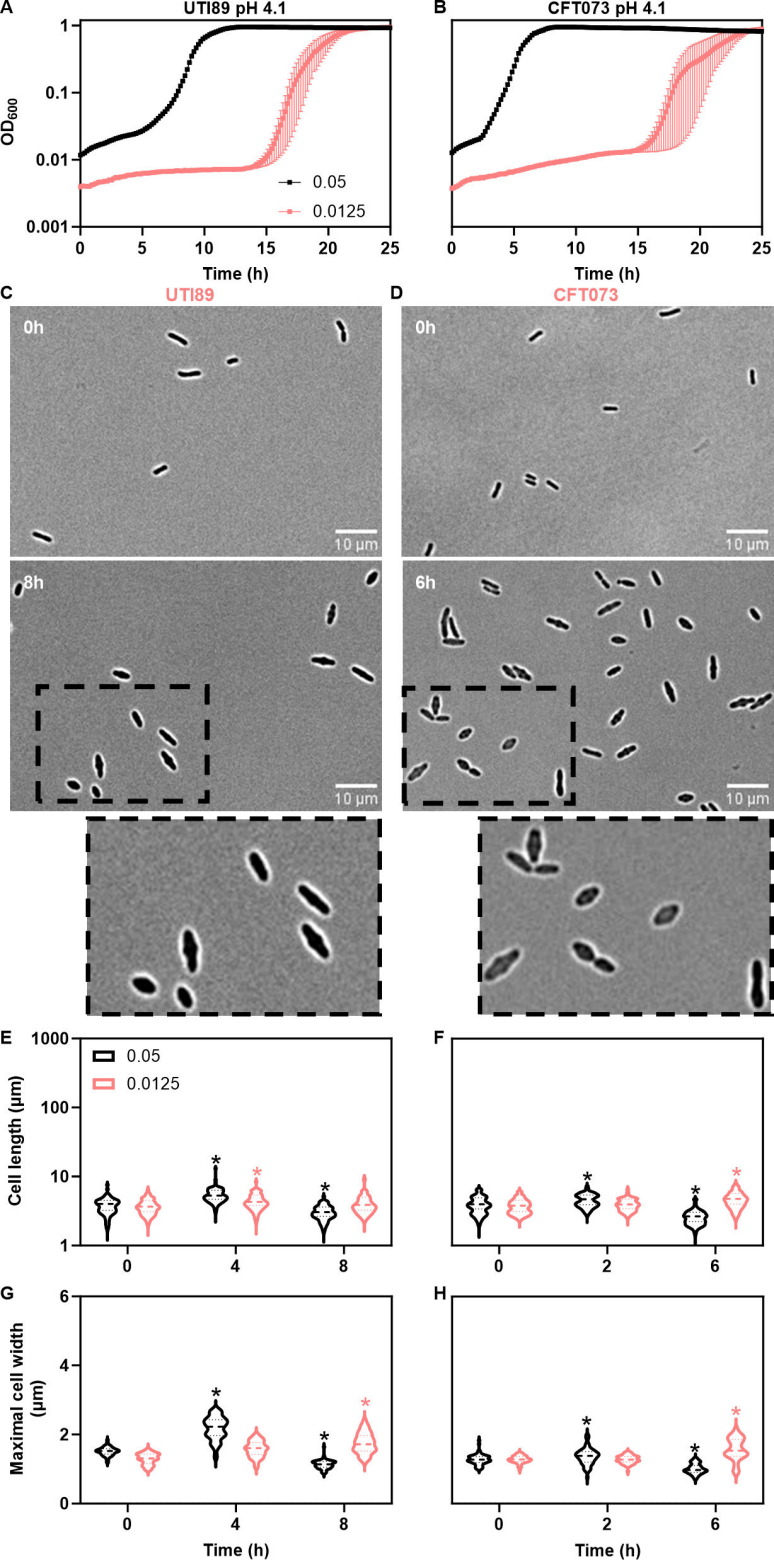
Uropathogenic *E. coli* strains exhibit cell density-dependent growth and morphology changes at near-lethal pH. Growth of *E. coli* strains UTI89 (**A**) and CFT073 (**B**) at indicated inoculum sizes in LB at pH 4.1. Mean and SEM of three replicates are shown. Morphology of UTI89 (**C**) and CFT073 (**D**) at indicated time points. Scale bar is 10 µm. Dotted lines specify the magnified area. Quantification of cell length for 100–150 cells of UTI89 (**E**) and CFT073 (**F**), and of maximal cell width for UTI89 (**G**) and CFT073 (**H**), done and represented as in Fig. 2. Mean and SEM of three biological replicates are shown. Significance of differences, assessed using a paired *t*-test, is indicated by asterisks (*P ≤ 0.05, **P ≤ 0.01).

To test whether other *E. coli* isolates also exhibit cell density-dependent multiphasic growth at near-lethal pH, we monitored the growth dynamics of two uropathogenic *E. coli* strains, UTI89 (18) and CFT073 (19) (Fig. 5; Fig. S9). Although these strains showed slightly higher acid tolerance than MG1655, the dependence of growth on cell density was observed for both strains at a pH value of 4.1 (Fig. 5A and B; Fig. S9A and B). However, the initial phase of the multiphasic growth was less pronounced, and in contrast to MG1655, these strains primarily exhibited biomass accumulation in the center of the cells and only minor elongation (Fig. 5C through H). Nevertheless, also for these strains, the resumption of the normal growth coincided with the restoration of the normal cell size and with an increase in pH (Fig. 5E through H; Fig. S9C and D).

Given this difference in the acid-induced phenotype between the K-12 and UTI89 and CFT073 strains, we further tested morphological changes for a number of strains from the *E. coli* Reference Collection (ECOR) of natural isolates of *E. coli* (20) grown in LB at pH 4.1. Both cell elongation and widening or their combination was observed in the ECOR strains belonging to phylogroups A (which also include *E. coli* K-12), B1, and D (Fig. S10A, B and D). Strains from the B2 phylogroup showed primarily cell widening (Fig. S10C), consistent with the phenotype of UTI89 and CFT073 that also belong to this group.

Because *Salmonella* and *E. coli* spp. share acid resistance mechanisms (2, 5, 21), we further tested growth of *S*. Typhimurium at conditions ranging from neutral to near-lethal acidic pH. We observed the growth rate reduction with lower initial medium pH, although at a lower pH than for *E. coli* strains, as well as the density dependence of growth (Fig. S11 and S12). The initial growth phase was less pronounced in *S*. Typhimurium than in *E. coli* K-12, but there was both a transient change in the cell size (Fig. S12C and D) and an increase in the medium pH (Fig. S12B) before resumption of normal division.

### Cross-protection between acid-tolerant and acid-sensitive strains in a mixed population

**Fig 6:**
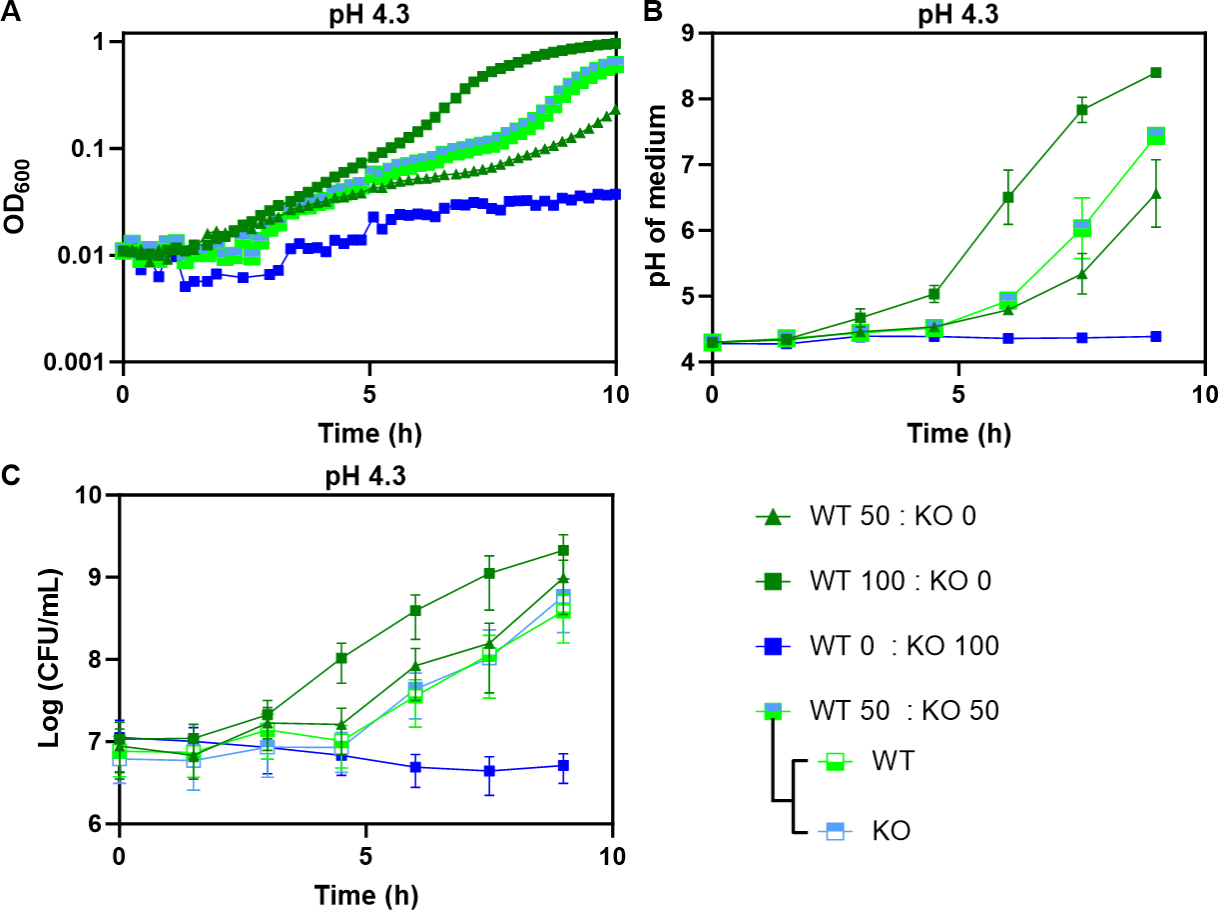
Acid-tolerant strain complements growth of an acid-sensitive strain in a coculture. Acid-tolerant (WT, MG1655 carrying pBAD24) and sensitive (KO, MG1655 *ΔcadC*) strains were cocultured at a total inoculum size of 0.05 in LB at pH 4.3. Two strains were mixed at equal proportions (50:50); monocultures were inoculated either at half the density or at full density, as indicated. (**A**) Growth of the cocultures. (**B**) Medium pH. (**C**) Colony-forming units (CFU) per milliliter of indicated cultures. The CFUs for WT and KO were distinguished by plating cocultures on LB agar with ampicillin and kanamycin, respectively. Mean and SEM of three biological replicates are shown.

Given our observation that *E. coli* growth at near-lethal pH relies on collective deacidification of the medium, we hypothesized that, in a coculture, acid-sensitive cells could be complemented for growth by acid-tolerant cells. To test for such cross-complementation, we cocultured wild type (WT) and Δ*cadC E. coli* MG1655 at equal amounts (WT 50:knockout [KO] 50) in LB at pH 4.3 and compared growth of this coculture to the monocultures of either WT or Δ*cadC* (Fig. 6). Consistent with our previous results (Fig. 4A), growth of the Δ*cadC* monoculture at low pH was severely reduced (Fig. 6A) and no deacidification of the medium was observed over 9 h of incubation (Fig. 6B). Overall growth and medium deacidification for the WT 50:KO 50 coculture was intermediate between the WT 100 (i.e., twice the WT density) and WT 50 monocultures, suggesting that Δ*cadC* cells contribute to medium deacidification, probably via other AR systems. Most importantly, quantification of the CFUs revealed a nearly identical growth of the WT and Δ*cadC* strains in the coculture at each time point, further confirming the collective nature of the acid tolerance under these conditions (Fig. 6C). Growth complementation was also observed in the coculture of WT 20:KO 80 (Fig. S13), demonstrating that even a minor fraction of acid-tolerant bacteria can support the growth of the entire population at low pH.

## DISCUSSION

Many neutralophilic bacteria, including pathogens, are naturally exposed to acidic environments such as the mammalian colon and acidified foods, soil, and water (2–6). To date, *E. coli* growth at near-lethal pH has only been studied for growth or no-growth outcomes (15, 22) or for the overall reduction in growth rate (4, 11, 12, 15). Here, we systematically examined the dependence of *E. coli* growth not only on pH but also on cell density, at growth-permitting, near-lethal acidic pH that is commonly encountered by *E. coli* in the acidic environments, including parts of the human gastrointestinal tract (7, 8), and thus important for its ecology.

We observed that *E. coli* growth at the near-lethal acidic pH is multiphasic, consisting of an initial phase of optical density increase, a mid-lag phase of apparent growth arrest, and a phase of growth resumption. This multiphasic growth was not solely determined by the medium pH, but also by the cell density of the culture. While the first and the third phases of growth showed little density dependence, low cell density cultures displayed higher duration of the mid-lag phase compared to high cell density cultures. Our results suggest that this density dependence could be explained by gradual collective deacidification of the medium during the mid-lag phase, which allows the resumption of growth above pH of 4.4. This deacidification depends not only on the number of deacidifying cells but also on the buffering capacity of the medium and on the presence of the AR systems. The non-growing, translation-inhibited *E. coli* cells could deacidify the medium similarly to the deacidification observed during the mid-lag phase of growth but only when pre-exposed to acidic pH before the inhibition of translation. In contrast, the deacidification of the medium by the translation-inhibited *E. coli* cells that were not pre-exposed to acidic pH was significantly slower. Thus, the ability of *E. coli* cells to deacidify the medium is enhanced by their pre-exposure to acidic pH, likely due to the induced expression of the AR systems.

We propose that, during the multiphasic growth of the culture, this induction of the AR systems occurs during the initial, density-independent phase of increase in the optical density. In *E. coli* MG1655, this first stage corresponded to an extensive (up to ~10-fold) cell elongation that could account for a similar-fold increase in OD. Consistent with the explanation that the increase in biomass during this stage is due to cell elongation in the absence of division, there was no increase, and even a moderate decrease, in the number of CFUs during the initial and the mid-lag phase of growth. The upper limit to this initial biomass increase is apparently set by the maximum elongation that is achievable by *E. coli* cells, as similar cell length was observed upon prolonged treatment with the cell-division inhibitor, cephalexin, in our experiments and those from others (23).

Elongation at low pH was recently reported for several *E. coli* strains and also for *Citrobacter rodentium,* but it was not observed for *Salmonella* (24). It was explained by the inhibition at low pH of the essential divisome protein FtsI (penicillin-binding protein 3) that is involved in septal peptidoglycan biosynthesis. In our experiments, changes in cell morphology were observed for all tested strains of *E. coli* as well as for *S*. Typhimurium. While some *E. coli* strains showed elongation similar to MG1655, other strains, particularly those from the B2 phylogroup that contains many uropathogens, increase in the cell width, suggesting that in those instances lateral rather than septal biosynthesis of peptidoglycan is inhibited first at low pH. Nevertheless, in all cases, also including *Salmonella*, an increase in the cell size correlating with the initial phase of optical density increase was observed. Our results indicate that this increase in biomass may be physiologically important as it is required to allocate additional protein resources in acid resistance at the initial stage of exposure to the near-lethal acidic pH. Notably, *E. coli* and *Salmonella* are known to transiently increase cell size in response to other stress conditions (25–27), indicating that the morphological response described here may be a general mechanism to enable bacteria to allocate resources in stress response.

Although our results suggest that subsequent exit from the mid-lag phase is primarily mediated by collective deacidification of the medium by *E. coli* culture, it might be further accelerated by selection for acid-tolerant mutations during prolonged incubation of the population under severe but growth-permissive acidity. However, the impact of such mutations appears to be modest, with only a minor reduction of the mid-lag phase duration in the culture that was previously passaged through growth at the near-lethal pH. Furthermore, the observed density-dependence duration of the mid-lag growth phase was well reproducible among replicates, suggesting that it was dominated by a deterministic process rather than by stochastic mutations.

The primary AR system involved in the observed density-dependent growth at the near-lethal acidic pH is the AR4 (Cad) system, as the lack of this system severely reduces the rate of the initial biomass accumulation and increases the duration of the mid-lag phase. Although the AR2 (Gad) system has been shown to make an important contribution to deacidification during acid shock by utilizing intracellular glutamate (10), our results suggest that it only plays an auxiliary role during the initial growth phase at near-lethal acid conditions. Nevertheless, the overexpression of the Gad system results not only in the increased initial growth rate but also in the reduced duration of the mid-lag phase. Since the conversion of glutamate to γ-aminobutyric acid and excretion of the latter by AR2 should not directly deacidify the external medium, an indirect deacidification by the sequestration of intracellular H^+^ can contribute significantly to the overall collective deacidification. This is also likely to be the case for other AR systems. Measurements of amino acid consumption and amine production suggest that the AR3 (Adi) system contributes to deacidification too, particularly at the later stage of growth. Moreover, the recently described mechanism that relies on serine deamination may also play a role (17).

The observed collective acid tolerance of *E. coli* growth at near-lethal pH may be related to the recently shown heterogeneous expression of the Cad system due to the low copy number of its regulator CadC (13, 28). It was suggested that stochastic differentiation of *E. coli* population into distinct *cad-on* and *cad-off* subpopulations (29) might represent division of labor in a bacterial population (13). Our results, demonstrating that even a minor fraction of Cad-positive cells is sufficient to enable growth of the entire population, support the feasibility of such division of labor.

Taken together, here we could identify collective dynamics and morphological changes that are important for *E. coli* growth under acidic conditions, contributing to understanding of the mechanisms that enable *E. coli* strains to invade acidic intestinal microenvironments (30–32). The observed response could be of general ecological significance and could also affect bacterial interactions with immune cells by counteracting phagocytosis (33) and/or by enabling survival within the acidic phagosome (30, 34).

## MATERIALS AND METHODS

### Bacterial strains and plasmids

All strains and plasmids used in this study are listed in Table S2. Genetically modified strains were derived from *E. coli* MG1655 using P1 transduction (35). pTrc99a-gadE was constructed by PCR amplification of *gadE* from the MG1655 genome followed by Gibson assembly to clone it into the pTrc99a expression vector inducible by isopropyl β-d-1-thiogalactopyranoside.

### Culture conditions

Bacteria were cultured in LB medium (10 g/L tryptone, 5 g/L yeast extract, and 5 g/L NaCl) at pH 7 (unless otherwise stated). HCl was used to adjust pH. Where indicated, lysine or other amino acids were added to a final concentration of 20 mM before adjusting the pH to 4.3. Pre-cultures were prepared by inoculating overnight culture at 1:50 dilution into fresh LB and incubating for ~1.5 h until the OD_600_ reached 0.50–0.70 at 200 rpm and 37°C. Pre-culture cells were washed three times (1,500 × *g*, 3 min, 20°C) using a medium with the target pH, and OD_600_ was adjusted to 0.50 using a 10 mm cuvette (REF 67742, Sarstedt). These OD-adjusted cultures were inoculated into LB with the target pH at 10.0% (OD_600_ = 0.05), 5.0% (OD_600_ = 0.025), 2.5% (OD_600_ = 0.0125), or 1.25% (OD_600_ = 0.00625) to generate inoculum sizes of ~1.0 × 10^7^ cells/mL, 5.0 × 10^6^ cells/mL, 2.5 × 10^6^ cells/mL, and 1.25 × 10^6^ cells/mL, respectively. HCl was utilized to acidify the medium. Growth was monitored in 48-well plates (500 µL/well, REF 677102, Greiner Bio-One) in a plate reader (Infinite, Tecan GmbH) using alternating cycles of 150 s linear (452 rpm) and orbital (218 rpm) shaking at 37°C. When indicated, homopiperazine-1,4-bis(2-ethanesulfonic acid) (AB117062, homo-PIPES, abcr GmbH) was added as buffer.

For pH measurements, the lids of 48-well plates were pierced with a sterile twisted drill bit to make sampling holes in each of the wells. The border of the plate and the holes in the wells were then sealed with parafilm to prevent evaporation. Samples were taken at specified intervals, and pH was measured using a standard pH meter (Thermo Scientific) or an Ultra-Micro-ISM pH meter (Mettler-Toledo) for small volumes. Quantification of CFUs was done by plating serial dilutions on LB agar plates containing the appropriate antibiotics and manual counting of colonies.

To test maximal cell elongation, pre-cultures grown as above were first transferred to LB medium at pH 7 and incubated at 200 rpm for 1 h at 37°C. Cells were then transferred to LB containing 100 µg/mL cephalexin at pH 7 and incubated at 200 rpm, 37°C for 1.5 h.

### pH changes by translation-inhibited cells

Pre-cultures grown as above were first transferred to LB medium with either pH 4.2 or 7.0 and incubated at 200 rpm for 1 h at 37°C. Cells were then transferred to LB containing 200 mg/mL chloramphenicol at pH 4.2 and incubated at 200 rpm, 37°C for 3 h. The OD_600_ was then adjusted to 0.50; the cultures were inoculated at the specified cell density; and OD_600_ and pH were monitored during incubation in the plate reader as described above.

### Bright-field microscopy

Cells were collected from 50 µL of culture by centrifugation (4,500 × *g*, 1 min, 20°C), and the supernatant was used for pH measurement. The pellet was immediately resuspended in 10 µL phosphate-buffered saline at pH 5.0. A 5 µL aliquot was placed on an agarose pad for microscopy. Images were captured with a ×20 objective using Zeiss Observer Z1 or a Zeiss Elyra 7. The length and width of 100–150 cells were measured using Fiji 1.53 segmented lines tool.

### Metabolite quantification via liquid chromatography–mass spectrometry

*E. coli* strain MG1655 was grown at an inoculum size of 0.025 in LB at pH 4.3 as described above. Every 5 h, samples were taken to quantify medium levels of amino acids and biogenic amines via liquid chromatography–tandem mass spectrometry as described before (36) and with the following specifications. Chromatographic separation was performed on an Agilent Infinity II 1290 HPLC system coupled to an Agilent 6495 ion funnel mass spectrometer. The injection volume was 1 µL. The mass spectrometer operated in positive electrospray ionization mode with the following conditions: electrospray ionization voltage of 2,000 V, nozzle voltage of 1,000 V, sheath gas at 250°C at 12 L/min, nebulizer pressure of 60 psig, and drying gas at 100°C at 11 L/min. Compounds were identified based on their mass transition and retention time using chemically pure standards. Further detailed settings are provided in Table S3. Chromatograms were integrated using MassHunter software (Agilent), and absolute concentrations were calculated based on an external calibration curve prepared in the sample matrix.

### Growth complementation assay

Cultures adjusted to OD_600_ = 0.50 were used to generate 50:50 and 20:80 cocultures of acid-tolerant and resistant strains at a total density of OD_600_ = 0.05 in LB pH 4.3. At each time point, serial dilutions were made and colonies were counted on LB agar plates containing the appropriate antibiotics.

### Statistical analysis and data plotting

Data analysis was performed using a paired *t*-test for comparisons of cell length over time and using unpaired *t*-test for comparisons between test groups. A *P* value below 0.05 was considered a significant difference. Statistical analysis and plots were performed using GraphPad Prism (version 10.1, San Diego, CA).

## Data Availability

All of the data are included in this article.

## SUPPLEMENTAL MATERIAL

Supplemental Material mbio.01932-25-s0001.docx Figures S1-S13 and Tables S1-S3.

ASM does not own the copyrights to Supplemental Material that may be linked to, or accessed through, an article. The authors have granted ASM a non-exclusive, world-wide license to publish the Supplemental Material files. Please contact the corresponding author directly for reuse.

## References

[1] Neidhardt FC, Curtiss R. 1996. Escherichia coli and Salmonella: cellular and molecular biology. 2nd ed. ASM Press, Washington, D.C.

[2] Schwarz J, Schumacher K, Brameyer S, Jung K. 2022. Bacterial battle against acidity. FEMS Microbiol Rev 46:fuac037. doi:10.1093/femsre/fuac037 35906711

[3] Guan N, Liu L. 2020. Microbial response to acid stress: mechanisms and applications. Appl Microbiol Biotechnol 104:51–65. doi:10.1007/s00253-019-10226-1 31773206 PMC6942593

[4] Kanjee U, Houry WA. 2013. Mechanisms of acid resistance in Escherichia coli. Annu Rev Microbiol 67:65–81. doi:10.1146/annurev-micro-092412-155708 23701194

[5] Lund P, Tramonti A, De Biase D. 2014. Coping with low pH: molecular strategies in neutralophilic bacteria. FEMS Microbiol Rev 38:1091–1125. doi:10.1111/1574-6976.12076 24898062

[6] Lin J, Smith MP, Chapin KC, Baik HS, Bennett GN, Foster JW. 1996. Mechanisms of acid resistance in enterohemorrhagic Escherichia coli. Appl Environ Microbiol 62:3094–3100. doi:10.1128/aem.62.9.3094-3100.1996 8795195 PMC168100

[7] Shalon D, Culver RN, Grembi JA, Folz J, Treit PV, Shi H, Rosenberger FA, Dethlefsen L, Meng X, Yaffe E, Aranda-Díaz A, Geyer PE, Mueller-Reif JB, Spencer S, Patterson AD, Triadafilopoulos G, Holmes SP, Mann M, Fiehn O, Relman DA, Huang KC. 2023. Profiling the human intestinal environment under physiological conditions. Nature 617:581–591. doi:10.1038/s41586-023-05989-7 37165188 PMC10191855

[8] Bown RL, Gibson JA, Sladen GE, Hicks B, Dawson AM. 1974. Effects of lactulose and other laxatives on ileal and colonic pH as measured by a radiotelemetry device. Gut 15:999–1004. doi:10.1136/gut.15.12.999 4448417 PMC1413067

[9] Lin J, Lee IS, Frey J, Slonczewski JL, Foster JW. 1995. Comparative analysis of extreme acid survival in Salmonella typhimurium, Shigella flexneri, and Escherichia coli. J Bacteriol 177:4097–4104. doi:10.1128/jb.177.14.4097-4104.1995 7608084 PMC177142

[10] Mates AK, Sayed AK, Foster JW. 2007. Products of the Escherichia coli acid fitness island attenuate metabolite stress at extremely low pH and mediate a cell density-dependent acid resistance. J Bacteriol 189:2759–2768. doi:10.1128/JB.01490-06 17259322 PMC1855797

[11] Xu Y, Zhao Z, Tong W, Ding Y, Liu B, Shi Y, Wang J, Sun S, Liu M, Wang Y, Qi Q, Xian M, Zhao G. 2020. An acid-tolerance response system protecting exponentially growing Escherichia coli. Nat Commun 11:1496. doi:10.1038/s41467-020-15350-5 32198415 PMC7083825

[12] Castanie-Cornet M-P, Penfound TA, Smith D, Elliott JF, Foster JW. 1999. Control of acid resistance in Escherichia coli. J Bacteriol 181:3525–3535. doi:10.1128/JB.181.11.3525-3535.1999 10348866 PMC93821

[13] Brameyer S, Schumacher K, Kuppermann S, Jung K. 2022. Division of labor and collective functionality in Escherichia coli under acid stress. Commun Biol 5:327. doi:10.1038/s42003-022-03281-4 35393532 PMC8989999

[14] Schumacher K, Brameyer S, Jung K. 2023. Bacterial acid stress response: from cellular changes to antibiotic tolerance and phenotypic heterogeneity. Curr Opin Microbiol 75:102367. doi:10.1016/j.mib.2023.102367 37633223

[15] Skandamis PN, Stopforth JD, Kendall PA, Belk KE, Scanga JA, Smith GC, Sofos JN. 2007. Modeling the effect of inoculum size and acid adaptation on growth/no growth interface of Escherichia coli O157:H7. Int J Food Microbiol 120:237–249. doi:10.1016/j.ijfoodmicro.2007.08.028 17961778

[16] Yang Y, M Pollard A, Höfler C, Poschet G, Wirtz M, Hell R, Sourjik V. 2015. Relation between chemotaxis and consumption of amino acids in bacteria. Mol Microbiol 96:1272–1282. doi:10.1111/mmi.13006 25807888 PMC5008178

[17] Wiebe MA, Brannon JR, Steiner BD, Bamidele A, Schrimpe-Rutledge AC, Codreanu SG, Sherrod SD, McLean JA, Hadjifrangiskou M. 2022. Serine deamination is a new acid tolerance mechanism observed in uropathogenic Escherichia coli. mBio 13:e0296322. doi:10.1128/mbio.02963-22 36468870 PMC9765748

[18] Chen SL, Hung C-S, Xu J, Reigstad CS, Magrini V, Sabo A, Blasiar D, Bieri T, Meyer RR, Ozersky P, Armstrong JR, Fulton RS, Latreille JP, Spieth J, Hooton TM, Mardis ER, Hultgren SJ, Gordon JI. 2006. Identification of genes subject to positive selection in uropathogenic strains of Escherichia coli: a comparative genomics approach. Proc Natl Acad Sci USA 103:5977–5982. doi:10.1073/pnas.0600938103 16585510 PMC1424661

[19] Welch RA, Burland V, Plunkett G, Redford P, Roesch P, Rasko D, Buckles EL, Liou S-R, Boutin A, Hackett J, Stroud D, Mayhew GF, Rose DJ, Zhou S, Schwartz DC, Perna NT, Mobley HLT, Donnenberg MS, Blattner FR. 2002. Extensive mosaic structure revealed by the complete genome sequence of uropathogenic Escherichia coli. Proc Natl Acad Sci USA 99:17020–17024. doi:10.1073/pnas.252529799 12471157 PMC139262

[20] Ochman H, Selander RK. 1984. Standard reference strains of Escherichia coli from natural populations. J Bacteriol 157:690–693. doi:10.1128/jb.157.2.690-693.1984 6363394 PMC215307

[21] Small P, Blankenhorn D, Welty D, Zinser E, Slonczewski JL. 1994. Acid and base resistance in Escherichia coli and Shigella flexneri: role of rpoS and growth pH. J Bacteriol 176:1729–1737. doi:10.1128/jb.176.6.1729-1737.1994 8132468 PMC205261

[22] Haberbeck LU, Oliveira RC, Vivijs B, Wenseleers T, Aertsen A, Michiels C, Geeraerd AH. 2015. Variability in growth/no growth boundaries of 188 different Escherichia coli strains reveals that approximately 75% have a higher growth probability under low pH conditions than E. coli O157:H7 strain ATCC 43888. Food Microbiol 45:222–230. doi:10.1016/j.fm.2014.06.024 25500388

[23] Hou S, Jia Z, Kryszczuk K, Chen D, Wang L, Holyst R, Feng X. 2020. Joint effect of surfactants and cephalexin on the formation of Escherichia coli filament. Ecotoxicol Environ Saf 199:110750. doi:10.1016/j.ecoenv.2020.110750 32446103

[24] López-Escarpa D, Castanheira S, García-Del Portillo F. 2022. OmpR and Prc contribute to switch the Salmonella morphogenetic program in response to phagosome cues. Mol Microbiol 118:477–493. doi:10.1111/mmi.14982 36115022 PMC9827838

[25] Zhang D, Yin F, Qin Q, Qiao L. 2023. Molecular responses during bacterial filamentation reveal inhibition methods of drug-resistant bacteria. Proc Natl Acad Sci USA 120:e2301170120. doi:10.1073/pnas.2301170120 37364094 PMC10318954

[26] Dev Kumar G, Macarisin D, Micallef SA. 2019. Salmonella enterica filamentation induced by pelargonic acid is a transient morphotype. Appl Environ Microbiol 85:e02191-18. doi:10.1128/AEM.02191-18 30446555 PMC6328770

[27] Mattick KL, Rowbury RJ, Humphrey TJ. 2003. Morphological changes to Escherichia coli O157:H7, commensal E. coli and Salmonella spp in response to marginal growth conditions, with special reference to mildly stressing temperatures. Sci Prog 86:103–113. doi:10.3184/003685003783238725 12838606 PMC10368319

[28] Ude S, Lassak J, Starosta AL, Kraxenberger T, Wilson DN, Jung K. 2013. Translation elongation factor EF-P alleviates ribosome stalling at polyproline stretches. Science 339:82–85. doi:10.1126/science.1228985 23239623

[29] Brameyer S, Hoyer E, Bibinger S, Burdack K, Lassak J, Jung K. 2020. Molecular design of a signaling system influences noise in protein abundance under acid stress in different gammaproteobacteria. J Bacteriol 202:e00121-20. doi:10.1128/JB.00121-20 32482722 PMC8404709

[30] Kittana H, Gomes-Neto JC, Heck K, Juritsch AF, Sughroue J, Xian Y, Mantz S, Segura Muñoz RR, Cody LA, Schmaltz RJ, Anderson CL, Moxley RA, Hostetter JM, Fernando SC, Clarke J, Kachman SD, Cressler CE, Benson AK, Walter J, Ramer-Tait AE. 2023. Evidence for a causal role for Escherichia coli strains identified as adherent-invasive (AIEC) in intestinal inflammation. mSphere 8:e0047822. doi:10.1128/msphere.00478-22 36883813 PMC10117065

[31] Kotlowski R, Bernstein CN, Sepehri S, Krause DO. 2007. High prevalence of Escherichia coli belonging to the B2+D phylogenetic group in inflammatory bowel disease. Gut 56:669–675. doi:10.1136/gut.2006.099796 17028128 PMC1942160

[32] O’Brien CL, Bringer M-A, Holt KE, Gordon DM, Dubois AL, Barnich N, Darfeuille-Michaud A, Pavli P. 2017. Comparative genomics of Crohn’s disease-associated adherent-invasive Escherichia coli. Gut 66:1382–1389. doi:10.1136/gutjnl-2015-311059 27196580

[33] Möller J, Luehmann T, Hall H, Vogel V. 2012. The race to the pole: how high-aspect ratio shape and heterogeneous environments limit phagocytosis of filamentous Escherichia coli bacteria by macrophages. Nano Lett 12:2901–2905. doi:10.1021/nl3004896 22591454

[34] Yu Y, Zhang Z, Walpole GFW, Yu Y. 2022. Kinetics of phagosome maturation is coupled to their intracellular motility. Commun Biol 5:1014. doi:10.1038/s42003-022-03988-4 36163370 PMC9512794

[35] Thomason LC, Costantino N, Court DL. 2007. E. coli genome manipulation by P1 transduction. Curr Protoc Mol Biol Chapter 1:1. doi:10.1002/0471142727.mb0117s79 18265391

[36] Scarinci G, Ariens J-L, Angelidou G, Schmidt S, Glatter T, Paczia N, Sourjik V. 2024. Enhanced metabolic entanglement emerges during the evolution of an interkingdom microbial community. Nat Commun 15:7238. doi:10.1038/s41467-024-51702-1 39174531 PMC11341674

