# Supplementary material for: Collective dynamics of Escherichia coli growth under near-lethal acid stress
Source: mBio. 2025 Sep 22;16(11):e01932-25. doi: 10.1128/mbio.01932-25 (PMC12607569; doi:10.1128/mbio.01932-25)
Supplement: Supplemental Material — Figures S1-S13 and Tables S1-S3. [file mbio.01932-25-s0001.docx]

**Supplemental Material**

**Collective dynamics of *Escherichia coli* growth under near-lethal acid stress**

Rafael R. Segura Munoz^1,2^ and Victor Sourjik^1,2, #^

^1^Max Planck Institute for Terrestrial Microbiology, 35043 Marburg, Germany

^2^Center for Synthetic Microbiology (SYNMIKRO), 35043 Marburg, Germany.

#Address correspondence to: Victor Sourjik, victor.sourjik@mpi-marburg.mpg.de

Running Head:

*E. coli* growth under near-lethal acid stress

**Contains:**

**Supplemental Figures S1-S13**

**Supplemental Tables S1-S3**

**Supplemental References**

**
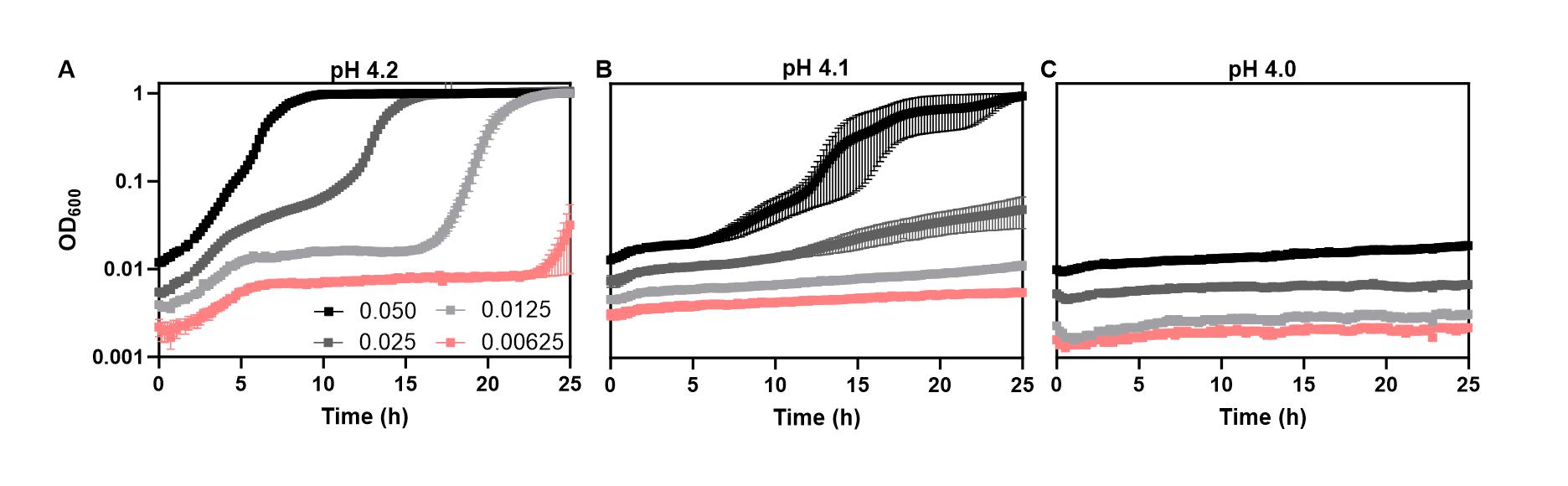
Fig. S1 *E. coli* exhibits cell density-dependent growth until pH 4.0** Growth of *E. coli* MG1655 at indicated inoculum sizes, measured as optical density at 600 nm (OD_600_) using plate reader, in unbuffered LB adjusted to initial pH of (A) 4.2, (B) 4.1 and (C) 4.0. HCl was used to adjust pH. Inoculum size values here and throughout correspond to OD_600_ measurements in a 10 mm cuvette using spectrophotometer, thus deviating from OD_600_ values measured in a plate reader. Mean and SEM of 3 biological replicates are shown. Difference between the density-dependence of growth at pH 4.2 in (A) from the data in Figure 1E is likely due to minor variation of the initial medium pH between experiments.

**
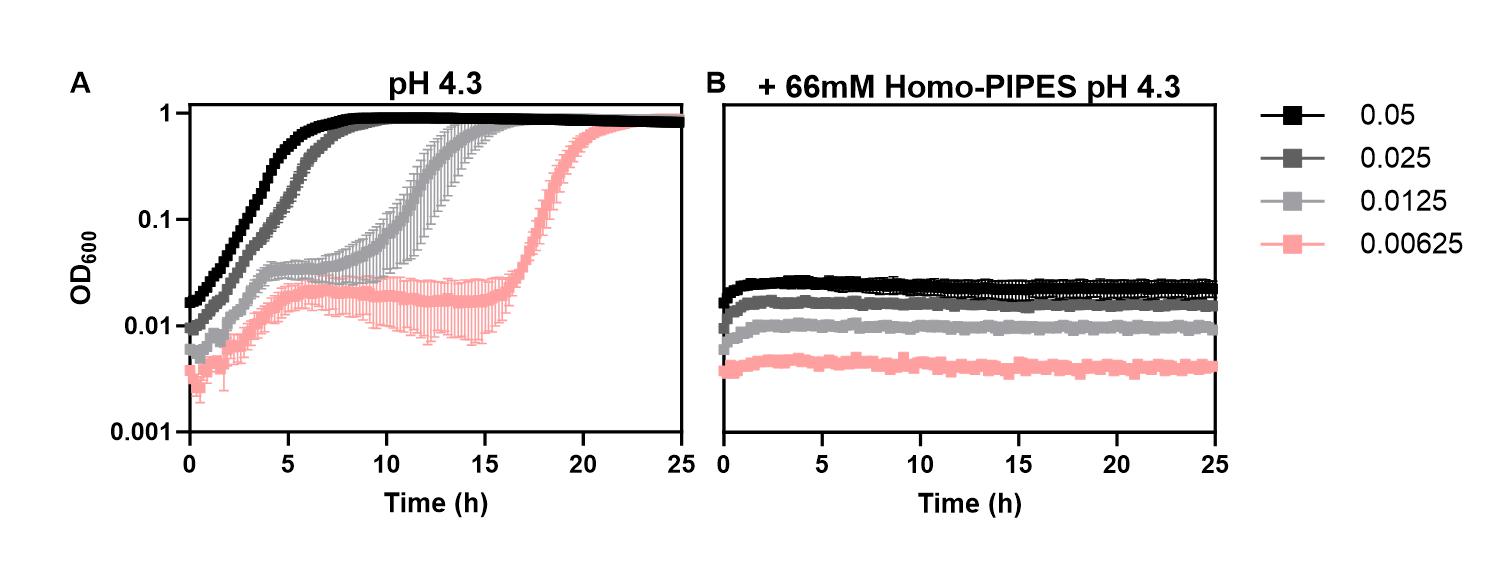
**

**Fig. S2 Comparison of *E. coli* growth in buffered and non-buffered medium.** Growth of *E. coli* MG1655 measured using plate reader at indicated inoculum sizes in LB set at initial pH 4.3 with (A) 0 mM and (B) 66 mM Homo-PIPES buffer. Mean and SEM of 3 biological replicates are shown.

**Fig. S3 Changes in cell size and medium pH during multiphasic *E. coli* growth.** *E. coli* MG1655 cultures at indicated inoculum size were grown in LB at pH 4.2. (A) Growth of the cultures with indicated inoculum sizes, measured using plate reader. (B) Medium pH for the same cultures, measured as described in Materials and Methods. Mean and SEM of 3 biological replicates are shown. (C) Quantification of cell length for 100-150 cells at indicated time points, with the distribution of responses, the average (dashed line) and the upper and lower quartiles (dotted lines) being indicated. Significance of difference compared to zero time point, assessed using a paired *t*-test, is indicated by asterisks (**p* ≤ 0.05).

**Fig. S4 Colony-forming unit (CFU) changes during multiphasic growth of *E.coli* at near-lethal acid pH**. *E. coli* MG1655 cultures at inoculum size 0.0125 were grown in LB pH 4.3 (A) and 4.2 (B), and corresponding CFU counts per mL of culture at pH 4.3 (C) and 4.2 (D). Mean and SEM of 3 biological replicates are shown.

**Fig. S5 Elongation of *E. coli* exposed to cephalexin.** *E. coli* MG1655 cultures at inoculum size 0.025 were grown in LB with 100 µg/mL cephalexin and pH 7.0. OD_600_ (A) and (B) cell length at indicated time points are shown. The distribution of cell length for 100-150 cells, the average (dashed line) and the upper and lower quartiles (dotted lines) are shown. Significance of difference compared to *t* = 0, assessed using a paired *t*-test, is indicated by asterisks (**p* ≤ 0.05).

**Fig. S6 Pre-exposure affects growth of *E.coli* at near-lethal acid pH.** *E. coli* MG1655 cultures at inoculum size 0.025 were grown in LB pH 4.2, regrown in LB pH 7.0, frozen-stored and re-tested in LB pH 4.2. Growth (characterized by OD_600_) of such pre-exposed cultures was compared to the original culture at inoculum size 0.025 in LB pH 4.2. Mean and SEM of 3 biological replicates are shown.

**Fig. S7** **Lysine supplementation increases growth rate at near-lethal acidic pH.** *E. coli* MG1655 cultures at inoculum size 0.05 were grown in LB pH 4.3 with or without supplementation with 20 mM lysine or glutamic acid, as indicated. Mean and SEM of 3 biological replicates are shown.

**Fig. S8 Deletion or overexpression of Gad system changes acid sensitivity.** *E. coli* MG1655 wild type and Δ*gadE* cultures at inoculum size 0.0125 were grown in LB pH 4.2 (A). Growth of pTrc99a and pTrc99a-*gadE* at inoculum size 0.0125 in LB pH 4.3 (B). Mean and SEM of 3 biological replicates are shown.

**Fig. S9** **Cell-density dependent growth in other *E. coli* strains.** *E. coli* strains UTI89 (A) and CFT073 (B) cultures at inoculum sizes 0.05 and 0.0125 were grown in LB pH 4.3. The medium pH change of UTI89 (C) and CFT073 (D) grown in LB pH 4.1, as shown in Figure 5. Mean and SEM of 3 biological replicates are shown.


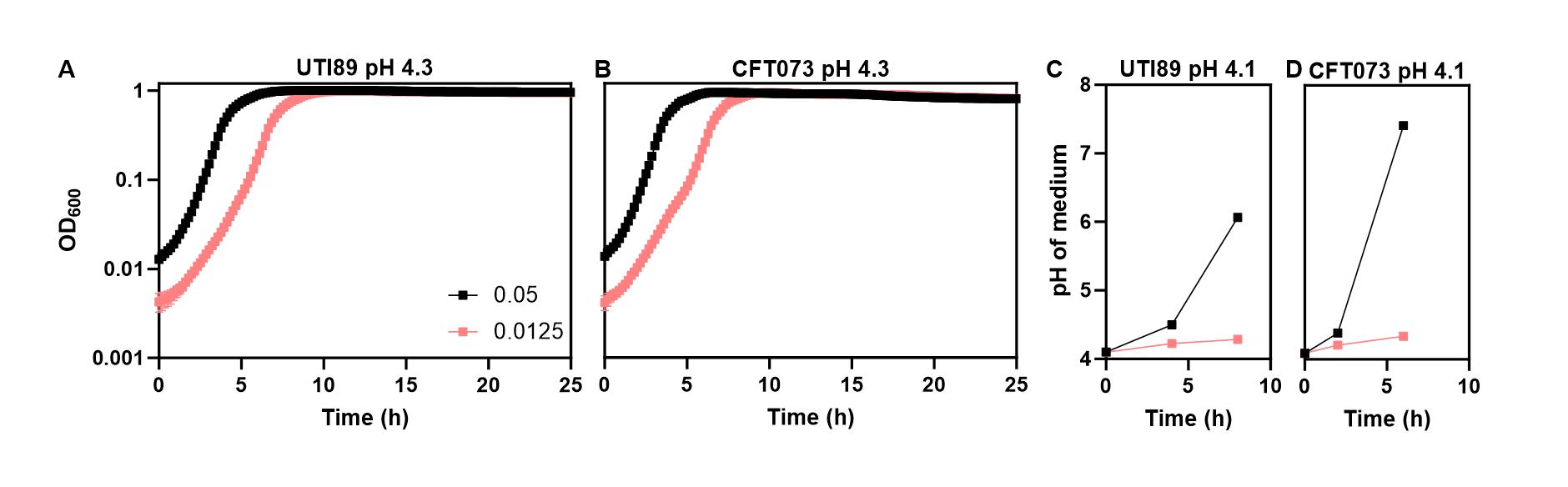


**Fig. S10 Cell shape of ECOR strains during** **growth in near-lethal acid pH.** *E. coli* strains from the *E. coli* ECOR collection representing the major phylogenetic groups were selected to grow in LB pH 4.1 during 10 hours. Morphology of strains *E. coli* ECOR 1, 2 and 10 of phylogenetic group A (A), strains *E. coli* ECOR 29, 33 and 69 of phylogenetic group B1 (B), strain *E. coli* ECOR 54, 60, 61, 62 and 63 of phylogenetic group B2 (C) and strain *E. coli* ECOR 36 of phylogenetic group D (D). Images were taken with a 40X objective. Scale bar is 10 µm. HCl was used to adjust pH.


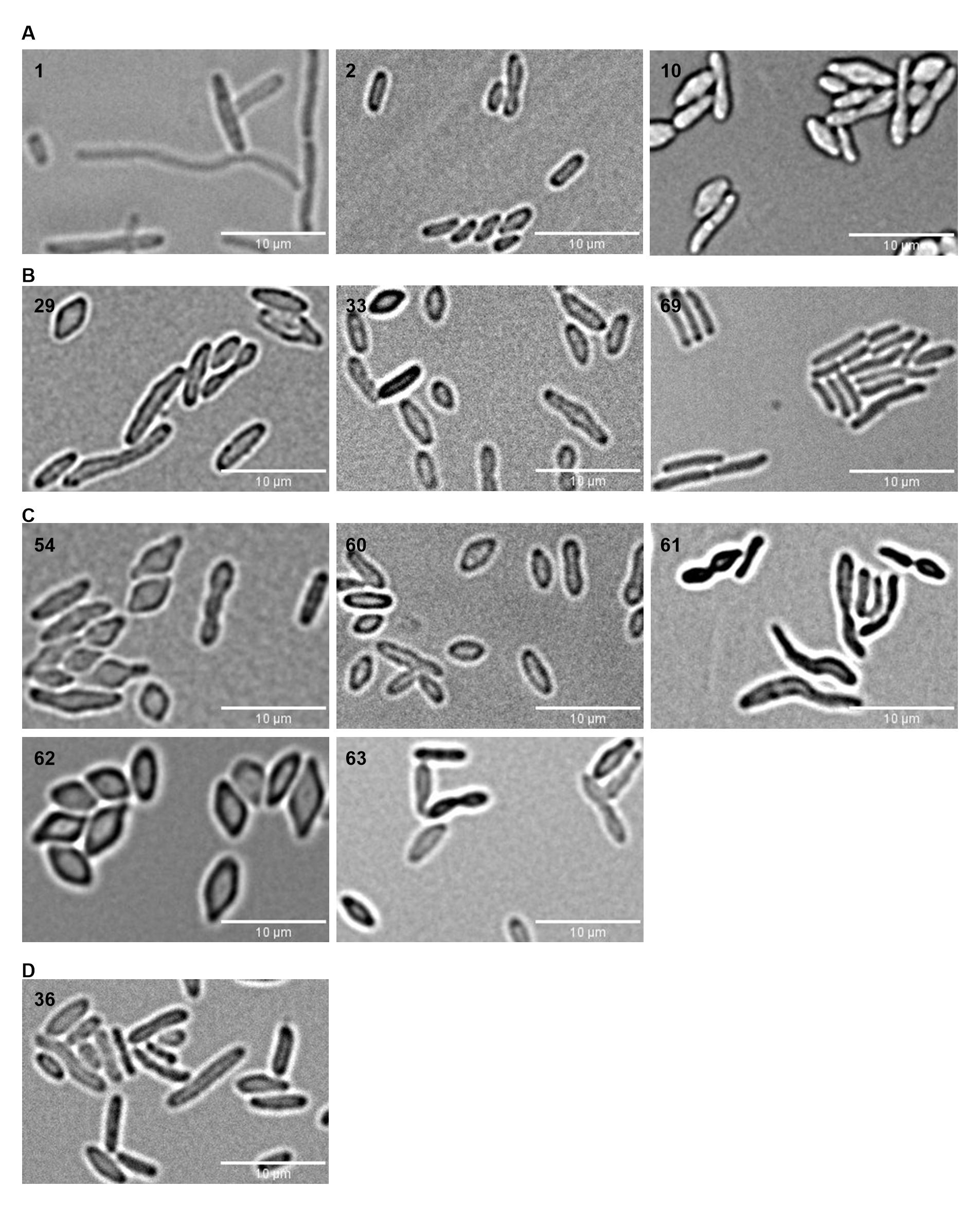


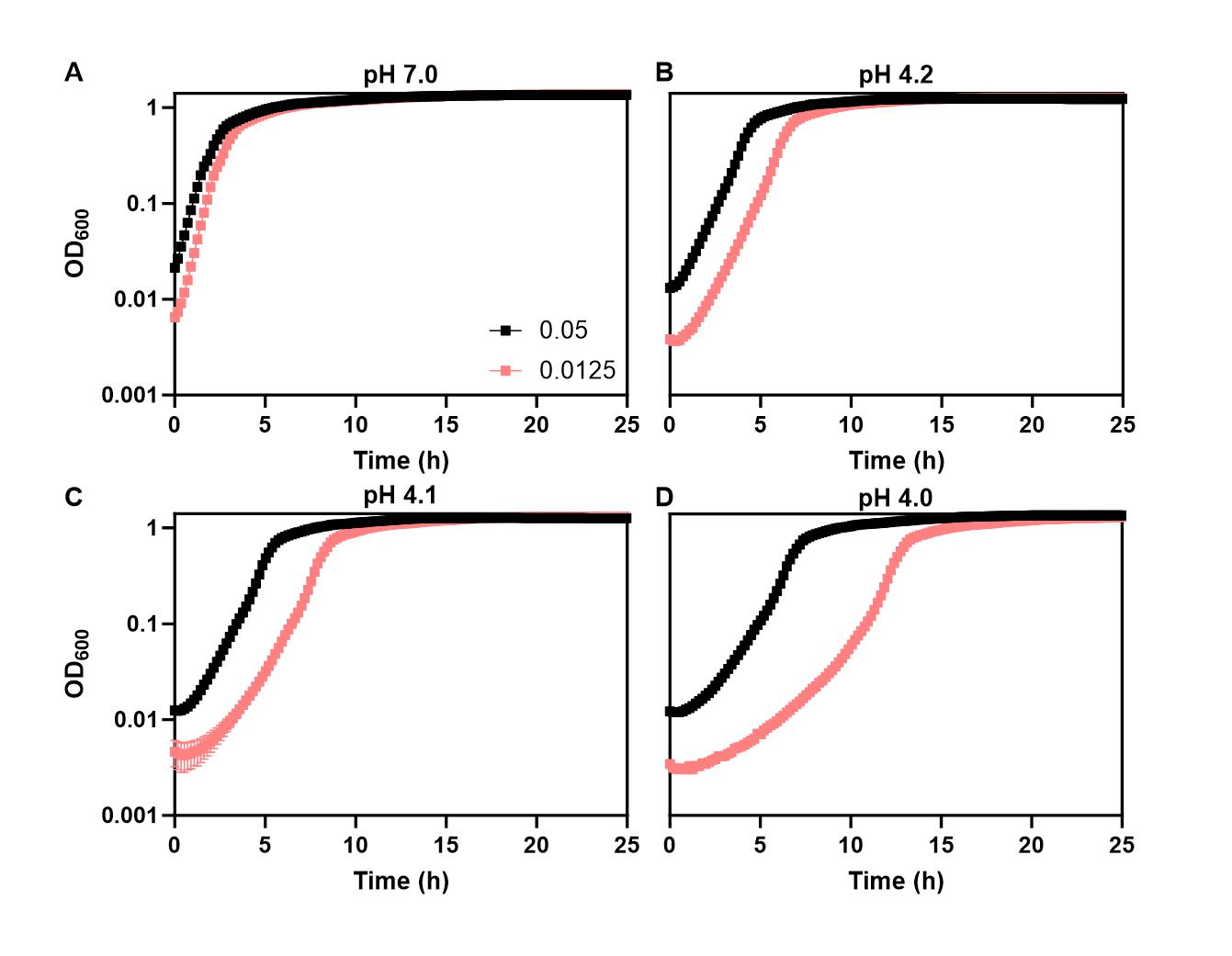


**Fig. S11 *Salmonella* growth at various pH.** *S.* Typhimurium cultures at inoculum sizes 0.05 and 0.0125 were grown in LB set at initial pH (A) 7.0, (B) 4.2, (C) 4.1 and (D) 4.0. Mean and SEM of 3 biological replicates are shown.

**Fig. S12 Multiphasic growth of *Salmonella* at pH 3.9.** *S.* Typhimurium cultures at inoculum sizes 0.05 and 0.0125 were grown in LB pH 3.9. (A) OD600nm and (B) medium pH. Mean and SEM of 3 biological replicates are shown. (C) Morphology of *E. coli* cells at indicated time points. Scale bar is 10 µm. (D) Quantification of cell length for 100-150 cells at indicated time points, with the distribution of responses, the average (dashed line) and the upper and lower quartiles (dotted lines) being indicated. Significance of difference compared to t=0, assessed using a paired *t*-test, is indicated by asterisks (**p* ≤ 0.05).

**Fig. S13 A minor fraction of acid-tolerant strain is sufficient to complement growth of acid-sensitive strain.** *E. coli* MG1655 WT and Δ*cadC* were cocultured at a 20:80 and 0.05 total inoculum size in LB pH 4.3. KO CFUs per mL are shown. Mean and SEM of 3 biological replicates are shown. SEM of the right bar is smaller than the line width. Significance of differences, assessed using an unpaired *t*-test, is indicated by asterisks (**p* ≤ 0.05)

| **TABLE S1. Medium pH after growth to stationary phase or after 25 hours of culture.** | | | | |
| --- | --- | --- | --- | --- |
|  | **Inoculum size (cells/mL)** | | | |
| **Medium** | 1.0×10^7^ | 5.0×10^6^ | 2.5×10^6^ | 1.25×10^6^ |
| LB pH 7.0 | 8.52 | 8.38 | 8.41 | 8.35 |
| LB pH 6.0 | 8.54 | 8.35 | 8.45 | 8.32 |
| LB pH 4.4 | 8.41 | 8.34 | 8.24 | 8.26 |
| LB pH 4.3 | 8.48 | 8.3 | 8.37 | 8.29 |
| LB pH 4.2 | 8.45 | 8.35 | 6.55* | 4.41* |
| LB+ Homo-PIPES pH 4.3 | 8.35 | 8.28 | 4.42* | 4.38* |
| *Cultures that have not reached saturation at the time of sampling. | | | | |

| **TABLE S2. Strains and plasmids utilized in this study.** | |  |
| --- | --- | --- |
| **Strain or plasmid** | **Relevant genotype or description^a^** | **Reference** |
| Bacteria | | |
| *E. coli* K-12 MG1655 | wild-type strain (F- lambda- *ilvG*- *rfb*-50 *rph*-1) | (1) |
| RRS123 | MG1655 derivative, Δ*cadC*; Km^R^ | This study |
| RRS124 | MG1655 derivative, Δ*gadE*; Km^R^ | This study |
| RRS125 | MG1655 derivative, Δ*adiY*; Km^R^ | This study |
| RRS109E | MG1655 derivative, pTrc99a-*gadE*; Amp^R^ | This study |
| RRS113E | MG1655 derivative, pBAD24-*cadC*; Amp^R^ | This study |
| *E. coli* UTI89 | wild-type strain | (2) |
| *E. coli* CFT073 | wild-type strain | (3) |
| *E. coli* ECOR 1 | natural isolate | (4) |
| *E. coli* ECOR 2 | natural isolate | (4) |
| *E. coli* ECOR 10 | natural isolate | (4) |
| *E. coli* ECOR 29 | natural isolate | (4) |
| *E. coli* ECOR 33 | natural isolate | (4) |
| *E. coli* ECOR 69 | natural isolate | (4) |
| *E. coli* ECOR 54 | natural isolate | (4) |
| *E. coli* ECOR 60 | natural isolate | (4) |
| *E. coli* ECOR 61 | natural isolate | (4) |
| *E. coli* ECOR 62 | natural isolate | (4) |
| *E. coli* ECOR 63 | natural isolate | (4) |
| *E. coli* ECOR 36 | natural isolate | (4) |
| *Salmonella enterica* Serovar Typhimurium ATCC 14028 | wild-type strain | (5) |
| Plasmids | | |
| pBAD24 | Expression vector with arabinose-inducible P_BAD_ promoter; pBR322 *ori*, Amp^r^ | (6) |
| pBAD24-cadC | *cadC_EC_* under control of arabinose-inducible promoter in pBAD24; Amp^r^ | (7) |
| pTrc99a | Expression vector with IPTG-inducible *trc* promoter; pBR ori; Amp^r^ | (8) |
| pTrc99a-gadE | gadE*_EC_* under control of IPTG-inducible promoter in pTrc99a; Amp^r^ | This study |

*^a^*Km^r^, kanamycin resistant, Amp^r^, ampicillin resistant.

| **TABLE S3. Parameter settings of all targets quantified using a LC-MS/MS.** | | | | | | | |
| --- | --- | --- | --- | --- | --- | --- | --- |
| **Compound** | **Precurser** | **Product** | **Dwell time** | **Fragmenter Voltage** | **Collision Energy** | **Cell Accelerator Voltage** | **Polarity** |
|  |  |  | **[msec]** | **[V]** | **[V]** | **[V]** |  |
| Tryptophane | 205.1 | 188 | 15 | 380 | 7 | 5 | Positive |
|  |  | 145.9 | 15 | 380 | 17 | 5 | Positive |
| Tyrosine | 182.1 | 165 | 15 | 380 | 6 | 5 | Positive |
|  |  | 136.1 | 15 | 380 | 12 | 5 | Positive |
| Arginine | 174.9 | 116 | 15 | 380 | 12 | 5 | Positive |
|  |  | 70.2 | 15 | 380 | 29 | 5 | Positive |
| Phenylalanine | 166.1 | 120.2 | 15 | 380 | 13 | 5 | Positive |
|  |  | 103.1 | 15 | 380 | 32 | 5 | Positive |
| Histidine | 156.1 | 110.1 | 15 | 380 | 16 | 5 | Positive |
|  |  | 83 | 15 | 380 | 30 | 5 | Positive |
| Methionine | 150.1 | 133 | 15 | 380 | 7 | 5 | Positive |
|  |  | 104 | 15 | 380 | 7 | 5 | Positive |
| Glutamate | 148.1 | 84.1 | 15 | 380 | 17 | 5 | Positive |
|  |  | 56.1 | 15 | 380 | 34 | 5 | Positive |
| Glutamine | 147.2 | 130.1 | 15 | 380 | 8 | 5 | Positive |
|  |  | 84.2 | 15 | 380 | 17 | 5 | Positive |
| Lysine | 147.1 | 130.1 | 15 | 380 | 8 | 5 | Positive |
|  |  | 84.1 | 15 | 380 | 19 | 5 | Positive |
| Aspertate | 134.1 | 88 | 15 | 380 | 9 | 5 | Positive |
|  |  | 74 | 15 | 380 | 14 | 5 | Positive |
| Asparagine | 133 | 87.1 | 15 | 380 | 17 | 5 | Positive |
|  |  | 74.2 | 15 | 380 | 16 | 5 | Positive |
| Isoleucine | 132.1 | 86.1 | 15 | 380 | 8 | 5 | Positive |
|  |  | 69.1 | 15 | 380 | 18 | 5 | Positive |
| Leucine | 132.1 | 86.1 | 15 | 380 | 8 | 5 | Positive |
|  |  | 30.3 | 15 | 380 | 18 | 5 | Positive |
| Threonine | 120.2 | 74.1 | 15 | 380 | 8 | 5 | Positive |
|  |  | 55.9 | 15 | 380 | 18 | 5 | Positive |
| Valine | 118.1 | 72 | 15 | 380 | 9 | 5 | Positive |
|  |  | 55.1 | 15 | 380 | 23 | 5 | Positive |
| Proline | 116 | 70 | 15 | 380 | 15 | 5 | Positive |
|  |  | 43.3 | 15 | 380 | 35 | 5 | Positive |
| Serine | 106.1 | 60.2 | 15 | 380 | 12 | 5 | Positive |
|  |  | 42.2 | 15 | 380 | 11 | 5 | Positive |
| Alanine | 90 | 44.1 | 15 | 380 | 12 | 5 | Positive |
| Glycine | 76.1 | 30.3 | 15 | 380 | 32 | 5 | Positive |
|  |  | 28.3 | 15 | 380 | 32 | 5 | Positive |
| Ornithine | 133.1 | 70 | 15 | 380 | 15 | 5 | Positive |
|  |  | 28.3 | 15 | 380 | 45 | 5 | Positive |
| Cadaverine | 103.2 | 86.2 | 15 | 380 | 8 | 5 | Positive |
|  |  | 69.2 | 15 | 380 | 17 | 5 | Positive |
| Putrescine | 89.1 | 89.1 | 15 | 380 | 0 | 5 | Positive |
| Agmatine | 131.1 | 114.1 | 15 | 380 | 9 | 5 | Positive |
|  |  | 72.2 | 15 | 380 | 16 | 5 | Positive |
| GABA | 104.1 | 104.1 | 15 | 380 | 0 | 5 | Positive |
|  |  | 70 | 15 | 380 | 15 | 5 | Positive |

**Supplemental References**

1. Blattner FR, Plunkett G, Bloch CA, Perna NT, Burland V, Riley M, Collado-Vides J, Glasner JD, Rode CK, Mayhew GF, Gregor J, Davis NW, Kirkpatrick HA, Goeden MA, Rose DJ, Mau B, Shao Y. 1997. The Complete Genome Sequence of *Escherichia coli* K-12. Science 277:1453–1462. https://doi.org/10.1126/science.277.5331.1453.

2. Chen SL, Hung C-S, Xu J, Reigstad CS, Magrini V, Sabo A, Blasiar D, Bieri T, Meyer RR, Ozersky P, Armstrong JR, Fulton RS, Latreille JP, Spieth J, Hooton TM, Mardis ER, Hultgren SJ, Gordon JI. 2006. Identification of genes subject to positive selection in uropathogenic strains of *Escherichia coli*: A comparative genomics approach. Proc Natl Acad Sci 103:5977–5982. https://doi.org/10.1073/pnas.0600938103.

3. Welch RA, Burland V, Plunkett G, Redford P, Roesch P, Rasko D, Buckles EL, Liou S-R, Boutin A, Hackett J, Stroud D, Mayhew GF, Rose DJ, Zhou S, Schwartz DC, Perna NT, Mobley HLT, Donnenberg MS, Blattner FR. 2002. Extensive mosaic structure revealed by the complete genome sequence of uropathogenic *Escherichia coli*. Proc Natl Acad Sci 99:17020–17024. https://doi.org/10.1073/pnas.252529799.

4. Ochman H, Selander RK. 1984. Standard reference strains of *Escherichia coli* from natural populations. J Bacteriol 157:690–693. https://doi.org/10.1128/jb.157.2.690-693.1984.

5. Tyler J, Chris S, A. GE, Howard O. 2010. Short-Term Signatures of Evolutionary Change in the *Salmonella enterica* Serovar Typhimurium 14028 Genome . J Bacteriol 192:560–567. https://doi.org/10.1128/jb.01233-09.

6. Guzman LM, Belin D, Carson MJ, Beckwith J. 1995. Tight regulation, modulation, and high-level expression by vectors containing the arabinose PBAD promoter. J Bacteriol 177:4121–4130. https://doi.org/10.1128/jb.177.14.4121-4130.1995.

7. Ude S, Lassak J, Starosta AL, Kraxenberger T, Wilson DN, Jung K. 2013. Translation Elongation Factor EF-P Alleviates Ribosome Stalling at Polyproline Stretches. Science 339:82–85. https://doi.org/10.1093/femsre/fuaa003.

8. Amann E, Ochs B, Abel K-J. 1988. Tightly regulated tac promoter vectors useful for the expression of unfused and fused proteins in *Escherichia coli*. Gene 69:301–315. https://doi.org/10.1016/0378-1119(88)90440-4.
